# Missed myocardial infarction in a vicenarian with malignant anomalous right coronary artery causing acute coronary syndrome: a case report

**DOI:** 10.1186/s13256-021-02770-0

**Published:** 2021-03-27

**Authors:** Nafeesah Ali, Kandace Baggan, Shari S. Khan, Paramanand Maharaj, Ronan G. Ali

**Affiliations:** 1 Department of Medicine, Eric Williams Medical Sciences Complex, St. Augustine, Trinidad and Tobago; 2 Adult Medicine Unit, Department Of Clinical Medical Sciences, University Of The West Indies, Bldg 67 2nd floor, Eric Williams Medical Sciences Complex, St. Augustine, Trinidad and Tobago

**Keywords:** Coronary anomaly, Myocardial infarction, Anomalous right coronary artery

## Abstract

**Background:**

Traditional coronary artery disease risk factors are well established and help risk stratify most patients presenting with chest pain syndromes. Young patients (under age 30 years) without other risk factors are thought to be at very low risk of coronary artery disease and acute coronary syndromes.

**Case presentation:**

We highlight the case of a 27-year-old Afro-Caribbean male who presented to hospital with chest pain and was discharged from the emergency room because he was thought to be low risk for ischemic heart disease. Laboratory investigations subsequently confirmed acute coronary syndrome. He was found to have an anomalous right coronary artery with a malignant origin running between the aorta and pulmonary artery eventually requiring surgical correction. Anomalous origins of the coronary arteries are rare causes of acute coronary syndromes, chest pain, and sudden cardiac death.

**Conclusion:**

Our patient could have easily had an adverse outcome as his diagnosis was missed by the initial treating physician. It is important to consider anomalous coronary artery origin in the evaluation of young symptomatic patients who may be otherwise low risk and not have traditional risk factors for ischemic heart disease.

## Introduction/background

Traditional coronary artery disease risk factors are well established and help risk stratify most patients presenting with chest pain syndromes. Young patients (under age 30 years) without other risk factors are thought to be at very low risk of coronary artery disease and acute coronary syndromes.

## Case presentation

A previously well 27-year-old Afro-Caribbean male nonsmoker presented to the emergency room following an episode of chest pain. The pain was central, burning, nonradiating, and lasted 40 minutes. It was rated 9/10 and was not associated with nausea or diaphoresis. He had been seen by a general practitioner 1 week prior with a similar complaint and diagnosed with gastroesophageal reflux disease (GERD). His past medical, family, and psychosocial history were noncontributory. He had undergone no prior medical or laboratory testing.

**Fig. 1:**
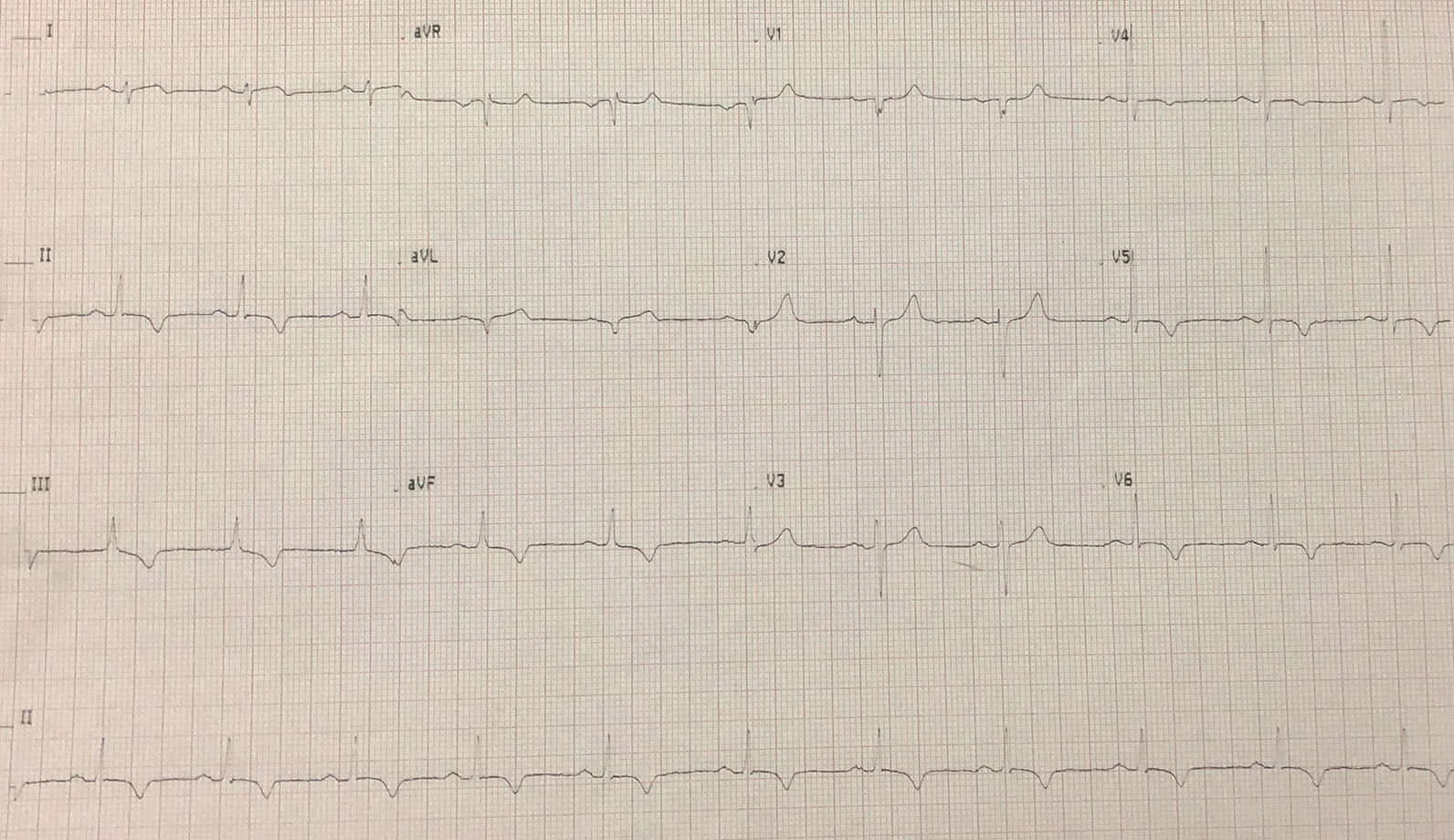
ECG on admission

On examination the patient was comfortable and pain free. Blood pressure was 148/56 mmHg, pulse 72 beats per minute, and oxygen saturation 99% on room air. The remainder of his physical examination was normal. Electrocardiography (ECG) showed T-wave inversions in leads II, III, aVF, V5, and V6 (Fig. 1). At this point, the patient wanted to go home as he was convinced it was another GERD episode and he felt better. Laboratory results had not yet been obtained, and the treating physician agreed to discharge the patient with a plan to return if symptoms recurred. Several hours later, the results were followed up and troponin I was found to be markedly positive with a value of 51 ng/mL (normal range 0–0.08 ng/mL). The patient was called and asked to return to hospital for admission.

**Fig. 2:**
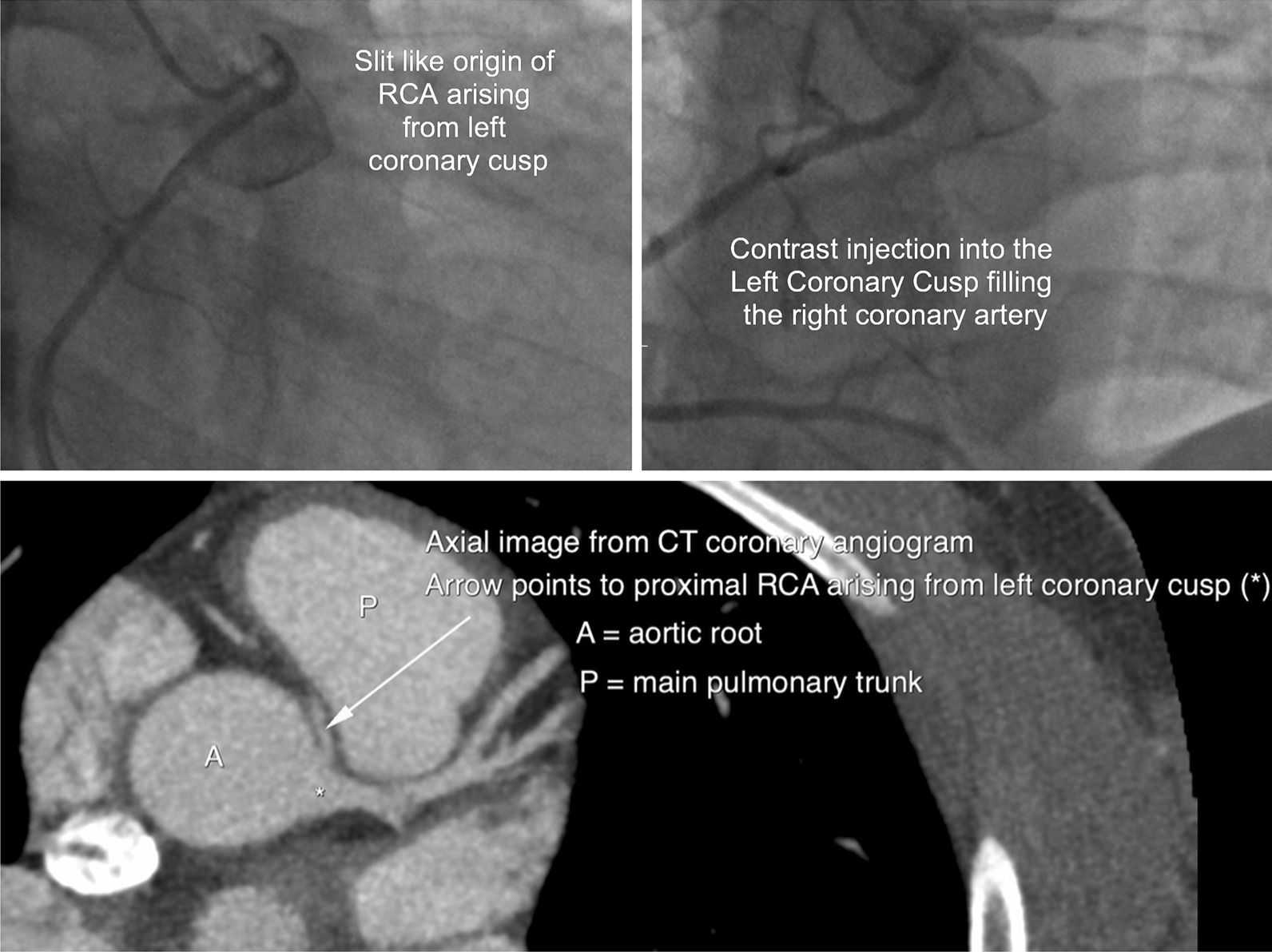
Multi-modality imaging of anomalous right coronary artery

Echocardiogram showed an ejection fraction of 65% without regional wall motion abnormality. Coronary angiography revealed an anomalous origin of the right coronary artery (RCA) from the left coronary cusp with an acute take-off angle creating an 80–90% ostial culprit lesion. Computerized tomography (CT) coronary angiogram confirmed that the RCA arose anomalously from the left coronary cusp and coursed between the pulmonary trunk and the aorta with a “malignant” angulation at its origin (Fig. 2). The patient was referred to cardiac surgery, and he underwent coronary artery bypass grafting (CABG) with a right internal mammary artery graft to the RCA. He had an uneventful postoperative course and recovery. At subsequent follow-up office visits, he was well and defaulted from the clinic 1 year after surgery. Two years postoperatively, he presented to the emergency room with atypical chest pain after doing heavy lifting at home. He underwent treadmill stress echocardiography, which was normal. His chest pain resolved with antiinflammatory medications, and he remains asymptomatic to date.

## Discussion

Coronary anomalies are found in 0.2–1.2% of the population [1]. The prevalence of an anomalous origin of the RCA (ARCA) arising from the left coronary cusp with interarterial course between the aorta and pulmonary artery varies between 0.026% and 0.25% [2]. An ARCA is more common than anomalous origin of the left coronary artery (ALCA), but ALCAs are more associated with sudden cardiac death [3]. An ARCA from the left coronary cusp with an interarterial course can increase risk of myocardial ischemia, arrhythmia, syncope, and sudden cardiac death [2, 3].

The exact pathophysiological mechanism remains unclear. There are several hypotheses [3]:

- Mechanical compression of the coronary artery between the pulmonary artery and aorta
- Acute take-off angle at the ostium of the RCA and left coronary sinus
- Abnormal slit-like RCA orifice in the aortic wall
- Anomalous RCA passage through the aortic commissure causing compression by the aortic valve commissure

The management of patients with anomalous coronary arteries is controversial. Sudden death occurs frequently in asymptomatic patients with ALCAs, so surgical repair is advised [3]. In asymptomatic ARCAs, sudden death is rare. It has been suggested in some studies that young patients (<35 years) with symptoms or ischemia should undergo surgery. In young patients without symptoms or ischemia, the best therapy is uncertain. Older patients without symptoms or ischemia do not need surgery [4].

Surgical treatment methods include the unroofing procedure, which manipulates and enlarges the orifice and creates an acute angulation that decreases the lateral compression of the intramural segment. This method does not manipulate the interarterial course and has good results [5]. Percutaneous coronary intervention relieves systolic compression, but selective cannulation and stent insertion in the anomalous RCA are difficult to perform because of the small, ectopic orifice and the long, curved intramural portion of the anomalous RCA [1]. Coronary artery bypass grafting is technically easy because it does not entail opening of the aorta or manipulation of the intercoronary commissure. Coronary reimplantation of the anomalous RCA in the right coronary sinus is also useful, but the disadvantage is that it can lead to neoostial stenosis [5].

## Conclusion

Because of our patient’s age and general good health, he was considered low risk for acute coronary syndrome and initially discharged home from the emergency room. Coronary artery anomalies are rare but important causes of acute coronary syndromes in young patients, many of whom will not have traditional risk factors for coronary artery disease.

## Data Availability

Data sharing is not applicable to this article as no datasets were generated or analyzed during the current study.

## Abbreviations

GERD: Gastroesophageal reflux disease
RCA: Right coronary artery
CABG: Coronary artery bypass grafting
ARCA: Anomalous right coronary artery
ALCA: Anomalous left coronary artery
